# Protocol to Exploit Waiting Resources for UASNs †

**DOI:** 10.3390/s16030343

**Published:** 2016-03-08

**Authors:** Li-Ling Hung, Yung-Jeng Luo

**Affiliations:** Department of Computer Science and Information Engineering, Aletheia University, New Taipei City 251, Taiwan; au4450@au.edu.tw

**Keywords:** bandwidth utilization, propagation delay, throughput, underwater acoustic sensor networks, UASNs

## Abstract

The transmission speed of acoustic waves in water is much slower than that of radio waves in terrestrial wireless sensor networks. Thus, the propagation delay in underwater acoustic sensor networks (UASN) is much greater. Longer propagation delay leads to complicated communication and collision problems. To solve collision problems, some studies have proposed waiting mechanisms; however, long waiting mechanisms result in low bandwidth utilization. To improve throughput, this study proposes a slotted medium access control protocol to enhance bandwidth utilization in UASNs. The proposed mechanism increases communication by exploiting temporal and spatial resources that are typically idle in order to protect communication against interference. By reducing wait time, network performance and energy consumption can be improved. A performance evaluation demonstrates that when the data packets are large or sensor deployment is dense, the energy consumption of proposed protocol is less than that of existing protocols as well as the throughput is higher than that of existing protocols.

## 1. Introduction

With the rapid development of sensor network technologies, underwater acoustic sensor networks (UASNs) have recently received increasing attention [1,2,3,4,5]. UASN technologies are used in various applications such as pollution monitoring, detection of submarines, and disaster warning. Because radio signals cannot be transmitted over a long distance in water, acoustic waveforms are used in underwater transmissions [4,5]. However, the propagation delay in UASNs is more significant than that in terrestrial wireless sensor networks. In general, the sound speed in the water is 1.5 km/s and the propagation delay is 0.67 s/km. Moreover, the maximum transmission distance is typically 1.5 km for a 10 kHz bandwidth. Thus, the one-hop neighbor distance is 1.5 km, and the propagation delay at this distance is approximately 1 s. In fact, the sound speed and maximum transmission distance both depend on the water column, temperature, and the center frequency of the signal. However, the speed varied does not affect the implementation of proposed mechanism because the mechanism considers the propagation delay time among sensors that is more practical while considering collisions for stably related propagation delays. In proposed mechanism, the sensors maintain the propagation delays of communicable neighbors via listening time information in the received packets. Long propagation delay leads to more complicated collision problems than those using radiofrequency methods [1,2,3,6]. Several methods have been proposed to solve collision problems in UASNs [3,7,8,9,10]. To avoid collisions, slotted mechanisms whereby each transmission should reserve a maximal propagation delay for receivers and senders have been proposed [3,10]. However, the wait time is significant due to the long propagation delay in water. Thus, bandwidth utilization and throughput in UASNs are much less than those in terrestrial wireless sensor networks.

## 2. Related Work

Research to improve bandwidth utilization and throughput in UASNs can be classified as temporal reuse, spatial reuse, and temporal and spatial reuse. Guo *et al.* [11] proposed the APCAP protocol that avoids collision by reserving multiple resources concurrently; however, their temporal reuse algorithm may reserve too many requests to efficiently exploit resources. Thus, more detailed resource scheduling is required. Xie *et al.* [12] proposed transmission opportunity scheduling for neighboring temporal reuse; however, their method assumes that sensors maintain the locations of neighbors, which can change periodically. In the protocol proposed by Noh *et al.* [5], each sensor maintains the propagation delay time of its two-hop neighbors, which allows transmitting sensors to avoid collisions. However, due to varying sensor topology in water, unslotted communication models cannot handle complicated communication. The propagation information can be used for temporal reuse but not spatial reuse. Diamant *et al.* [13] proposed topology-transparent scheduling for spatial reuse that uses reliable topology information, which requires significant resources to maintain. Ng *et al.* [4] proposed the reverse opportunistic packet appending (ROPA) protocol, which uses the idle bandwidth of senders in the period between sending RTSs and receiving CTSs. However, ROPA does not handle communications from other neighbors and does not use the receivers’ waiting resources. Ng *et al.* [14] also proposed a bidirectional concurrent MAC protocol to increase channel utilization. The sensors of a sender-receiver pair can transmit packets concurrently after each handshake. However, this transmission model may be unsuitable for general communication environments.

To improve throughput, researchers have proposed exploiting bandwidth waiting for propagations by considering temporal and spatial reuse. Ma *et al.* [15] proposed a TDMA-like scheduling algorithm using the approximate ratios of weighted traffic load due to interference awareness. However, the TDMA-like algorithm may be unsuitable for resource constrained wireless networks. Diamant *et al.* [16] proposed a handshake scheduling protocol to avoid collisions by considering spatial-temporal reuse; however, application is limited to stationary networks. Liao *et al.* [17] proposed a receiver-initiated protocol to decrease packet delay and increase throughput. However, for complicated neighbor relationships in UASNs, considering only receivers may be insufficient. Chen *et al.* [18] proposed the CS-MAC protocol. In CS-MAC, sensors do not send more control packets to negotiate but send data packets directly after determining that the packet will arrive at the receiver before the negotiated packet. However, CS-MAC assumes that the data packet transmission time is less than the propagation time between two packets such as an RTS/CTS pair and a CTS and its data packet. Basagni *et al.* [19] analyzed transmission performance with different packet sizes. To reduce energy consumption in UASNs, larger packets are considered better. Due to long propagation delay, large packets are more efficient than multiple small packets. In UASNs, sensing information can include large amounts of monitoring data. Moreover, to reduce the effect of long propagation delay, the number of transmissions should be reduced as much as possible. Thus, data should be collected and then transmitted when the amount of data is sufficient; thus, a large packet size may be more suitable for UASNs.

## 3. System Model and Problem Formulation 

### 3.1. System Model

**Figure 1:**
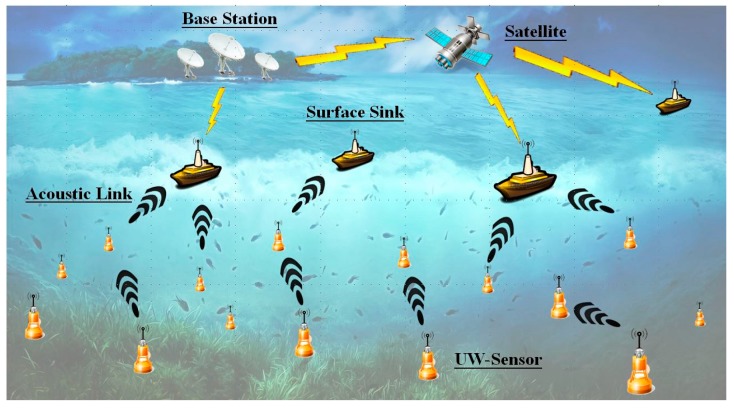
Underwater sensors transmit data to sinks on the surface.

A set of *h* sensors G = {*n*_1_, *n*_2_,…, *n_h_*} are distributed underwater. The sensors must transmit sensing information to surface sinks via multi-hop transmission. Thus, sensors at greater depths transmit packets to sensors closer to the surface, as shown in Figure 1. The proposed protocol is based on a slotted model. UASN synchronization mechanisms have been proposed [20,21,22], and we assume that the sensors in the water are synchronized. Moreover, sensor locations can change with water currents. In proposed mechanism, the propagation delay between sensors is more important than the locations of sensors. We assume that the localization problem has been deal with by other protocols [23,24]. Thus, in proposed model, the sending time stamp is included in each sent packet, and we assume all control packets including RTS, CTS, and Ack are the same size. Given the long propagation delay in water, a large data packet may be required as this can reduce the number of transmissions. Although the transmission problem in the water includes the transmission rate and the related distance, the propagation delay is according to the distance between sensors and the sound speed in the water. Moreover, the collision occurs when the two or more packets arrived at a sensor at the same time. Those packets are sent by the neighbors and arrive at the sensor after the relative propagation delaying time. Thus, we only focus on the propagation delay. Moreover, in our environment, the acoustic network is considered as a multi-hop manner half-duplex modern with an omnidirectional antenna. Each sensor maintains a list of one-hop neighboring propagations.

EW-MAC is a slotted-based protocol. Let *τ_max_* and *ω* denote maximal propagation delay time and time required to transmit a control packet, respectively. In EW-MAC, the duration of each time slot is *τ_max_* + *ω*. To avoid collisions, each packet, either a control or data packet, is sent at the beginning of a time slot. To balance fairness, each RTS packet includes a random priority value *rp* related to the contention and wait times of the sending sensor. When a receiver receives multiple RTS packets, it selects the sender with the highest *rp*.

### 3.2. Problem Formulation

Our objective is to develop a MAC protocol that improves throughput and energy consumption in UASNs. First, antennas and communication constraints should be described. Let *Pkt^t^_s_*_,*r*_ be the transmitting state of packet *Pkt_s,r_* at time *t*. *Pkt^t^_s_*_,*r*_ equals 1 when sensor *s* transmits a *Pkt_s,r_* to its one-hop neighbor *r* at time *t*. In addition, |*Pkt*| represents the time required to transmit packet *Pkt*. Given antenna characteristics, a sensor cannot transmit and receive simultaneously. With EW-MAC, the antenna remains in the receive state when it is not transmitting. Furthermore, to avoid interfering with communicating neighbors, messages from other neighbors should not arrive at the sensor when the sensor is receiving its messages. While a sensor is transmitting, its neighbors are not able to receiving from other sensors when its message arrives at them. Let *R_Pkt^t^_s,r_* represent the transmission result of *Pkt^t^_s,r_*, if *r* receives *Pkt^t^_s,r_* from *s* without interfered by its neighbors, then the value of *R_Pkt^t^_s,r_* is 1. If *r* does not receive the *Pkt^t^_s,r_* successfully, then the value of *R_Pkt^t^_s,r_* is 0. Therefore, the result of transmission from sensors *s* to *r* can be denoted by the following situations shown as Equation (1),

(1) $$R\_Pkt_{s,r}^{t}=\prod_{l=0}^{l=|Pkt_{s,r}^{t}|}(Pkt_{s,r}^{t+l}\wedge \;!Pkt_{r,x}^{t+l+\tau_{sr}}\wedge \;!Pkt_{z,y}^{t+l+\tau_{sr}-\tau_{zr}}),\;r,\;s,\;x,\;y,\;z\in G,\;\forall x,\;z\in N(r),\;y\in N(z)$$

where *t* is the start time for sensor *s* sending a packet and *τ_sr_* is the propagation delay between sensors *s* and *r*. *N*(*r*) denotes the set of one-hop neighbors from *r*. In addition, ∧ and ! denote logical AND and NOT, respectively. If *a* and *b* equal 0 and 1, respectively, then !*a* and !*b* equal 1 and 0. When *s* sends *Pkt^t^_s,r_* to *r* at time *t*, and no packets which are sent by the one-hop neighbors of *r* arrive at *r* while *r* is receiving *Pkt^t^_s,r_,* *R_Pkt^t^_s,r_* equals 1. If any other packet arrives at *r* during time *t* and *t* + |*Pkt^t^_s_*_,*r*_| + *τ_sr_*, then *R_Pkt^t^_s,r_* equals 0. In other words, when *r* successfully receives *Pkt^t^_s,r_* which is sent by *s* at time *t*, sensor *r* has not sent any packet and no packet from other neighbors arrived at *r* during the receive period (*t +* *τ_sr_*, *t* + |*Pkt^t^_s,r_*| + *τ_sr_* ). The proposed mechanism does not fix the packet size for its flexibility. Thus, |*Pkt^t^_s_*_,*r*_| is a variable which represent the time period required to transmit packet *Pkt^t^_s_*_,*r*_. Therefore, the amount of successfully received data for sensor *k* during time duration *T* is represented by *dr_k_*, that obtained by Equation (2).

(2) $$dr_{k}=\sum_{t=0}^{T}\sum_{s=n_{1}}^{n_{h}}\left(R\_Pkt_{s,k}^{t}\times |Pkt_{s,k}^{t}|\times BW\right)$$

where *BW* is the given transmission amount of a sensor in each time unit for the environment. 

The main purpose of this study is to improve throughput and energy consumption. Assume that the throughput of Algorithm A, $TP_{A}^{T}$, is evaluated by the amount of data successfully received during *T*. Thus, $TP_{A}^{T}$ is defined by Equation (3).

(3) $$TP_{A}^{T}=\frac{\sum_{k=n_{1}}^{n_{h}}dr_{k}}{T}$$

where *dr_k_* is the amount of data that sensor *k* successfully receives during *T*. The total energy consumption of sensors in the network using Algorithm A during *T* is defined as *PC_A_^T^*, where *E^A^* denotes the efficiency index of Algorithm *A.* An efficient algorithm should facilitate higher throughput and reduce energy consumption. Thus, we attempt to maximize $TP_{A}^{T}$ and minimize energy consumption for all sensors in the network *PC_A_^T^*. The efficiency index is defined by Equation (4).

(4) $$E_{A}=\frac{TP_{A}^{T}}{PC_{A}^{T}}$$

Table 1 lists the notations in this study.

## 4. Proposed EW-MAC Algorithm

**Figure 2:**
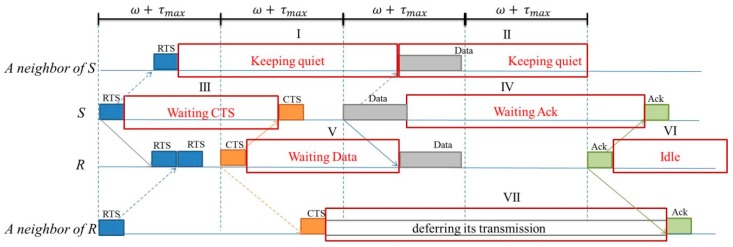
Wait periods that can be utilized in underwater transmissions.

The proposed EW-MAC is a four-way handshake communication protocol for a communication pair. Each successful communication is achieved by transmitting RTS, CTS, Data, and Ack packets. If sensor *i* receives a control packet from a neighbor, *i* knows the destination of the packet, the propagation delay between the negotiating pair, and the propagation delay between the sender and itself. Since sensors know the packet destination and propagation delay among related sensors, they can utilize the idle time of neighbors to transmit extra messages. For example, Figure 2 shows the idle periods that can be utilized, *i.e.*, blocks I–VII. Periods I and II represent the time that can be used for sensors near a negotiating and negotiated sender. Periods III and IV represent the time that can be exploited for a negotiating or negotiated sender. In EW-MAC, idle periods can be used to negotiate extra communications. To avoid interfering with neighbors, extra communications are allowed when the extra packets can use periods I and III, periods I and IV, or periods II and IV. The Figure 2 is an example of successful communication. We do not assure that there is no collision between RTS packets. When the RTS packets received in R from S collides with the RTS from others, the later communications in Figure 2 will not occur. The following describes the EW-MAC protocol in detail. Section 4.1 describes the design of EW-MAC, which exploits unused waiting resources. Section 4.2 describes how EW-MAC requests extra communication. Section 4.3 introduces the initialization and information maintenance of neighbor sensors. To efficiently exploit waiting resources (idle bandwidth and available sensors), sensors collect the information of one-hop neighbors, such as propagation delay between one-hop neighbors. 

### 4.1. EW-MAC Design and Assumptions

The proposed EW-MAC follows the IEEE802.11 four-way handshake communication model. EW-MAC is based on a slotted and synchronized UASN environment. Each communication is negotiated by an RTS/CTS pair. A sensor keeps quiet when it knows that neighbors are communicating. Due to the long propagation delay in underwater acoustic transmission, sensors transmit their packets at the beginning of a time slot. Note that duration of a time slot is equal to the propagation time to the maximum distance neighbors in water. The duration of time slot |*ts*| is *ω* + *τ*_max_, where *τ*_max_ is the maximum propagation delay and *ω* is the duration of transmitting a control packet. In the proposed protocol, when sensor *a* intends to communicate with a neighbor but receives a negotiation packet from a neighbor for another sensor after it sends the neighbor an RTS, *a* fails to contend for communication. The EW-MAC protocol provides an extra communication chance when a sensor fails to contend for communication. The extra communication chance is permitted when the neighbor knows that extra transmissions will not interfere with its negotiated transmissions. In our environment, the acoustic network is considered as a multi-hop manner half-duplex modern with an omnidirectional antenna. Each sensor maintains a list of one-hop neighboring propagation delays. The propagation delay between each two neighbors is varied. In proposed mechanism, the time stamp is appended to each sent packet. The sensors maintain the propagation delays of neighbors via calculating the difference of sent time and arrived time of each receiving packet. 

#### Negotiation for Communication in UASN

When sensor *s* intends to transmit data to a neighbor (e.g., sensor *r*), sensor *s* sends an RTS packet to *r* at the beginning of a time slot, e.g., the *t*th time slot. The RTS arrives at *r* within this same time slot. If *r* can receive data from *s*, *r* replies with a CTS packet at the beginning of the (*t* + 1)th time slot. By this condition, *s* and *r* form a pair of negotiated sensors. Thus, *s* sends the data packet to *r* at the beginning of the (*t* + 2)th time slot, and *r* replies with an Ack packet at the beginning of the next slot after it finishes receiving the data. To increase communication fairness, each RTS has a priority value according to the wait time of the sending sensor. When a receiver receives more than one RTS, it chooses the one with the greatest priority value.

Here, *Pkt_s_*_, *r*_ represents sensor *s* transmitting *Pkt* to sensor *r*. For example, *RTS_a_*_,*b*_ indicates that sensor *a* sends an RTS to sensor *b*. *Pkt*(*ts, s*, *r*) represents whether sensor *s* sends *Pkt* to sensor *r* at the *ts*th time slot; if yes, *Pkt*(*ts, s*, *r*) equals 1. Otherwise, *Pkt*(*ts, s*, *r*) equals 0. For example, *RTS*(*t, a*, *b*) equals 1 if sensor *a* sends an RTS to sensor *b* at the *t*th time slot. In addition, let *ts*(*Pkt_s_*_,*r*_) represent the time slot when sensor *s* sends a *Pkt* to sensor *r*. Thus, if *ts*(*Pkt_s,r_*) equals *t*, then *Pkt*(*t, s*, *r*) equals 1. For example, if sensor *a* sends an RTS to sensor *b* at the 7th time slot, then *ts*(*RTS_a_*_,*b*_) equals 7 and *RTS*(7*, a*, *b*) equals 1. Note that if *RTS*(*t, s*, *r*) equals 1 and *CTS*(*t* + 1*, r*, *s*) equals 1, then communication between sensors *s* and *r* is negotiated. Moreover, it is expected that *Data_s_*_,*r*_ will be transmitted at the (*t* + 2)th time slot. Since the data packet size in the proposed protocol is flexible, the time slot for sensor *r* transmitting *Ack_r_*_,*s*_ is derived by Equation (5),

(5) $$ts(Ack_{r,s})=ts(Data_{s,r})+\left\lceil \frac{TD_{s,r}+\tau_{s,r}}{\left|ts\right|}\right\rceil$$

where *TD_s,r_* is the transmission time for *Data_s_*_,*r*_ and (*TD_s,r_* + *τ_s,r_*)/|*ts*| is the number of time slots for *r* receiving *Data_s_*_,*r*_.

**Figure 3:**
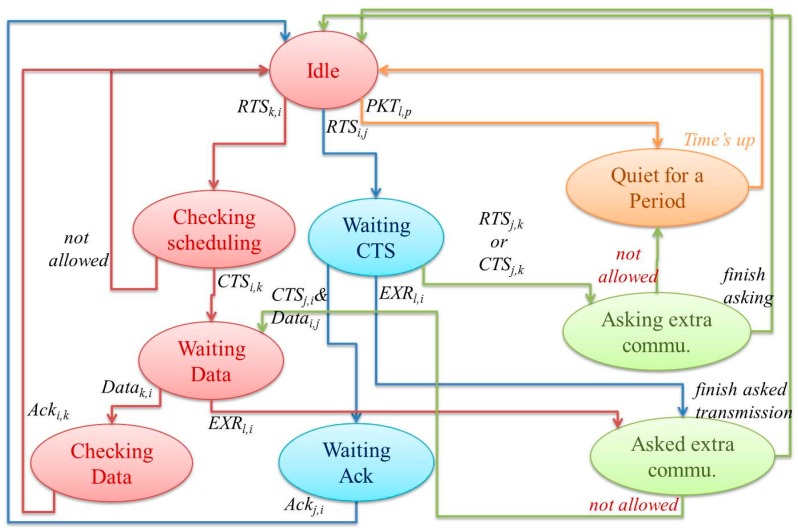
State transfer diagrams of sensor *i.*

Figure 3 shows the state transfer diagram of a sensor. Figure 3 shows the state transfer of sensor *i*, where sensors *j*, *k*, and *l* are the neighbors of *i*. While initializing, the state of *i* is “Idle.” The state of *i* can change from “Idle” to “Quiet”, “Checking Scheduling”, or “Waiting CTS” states. If sensor *i* receives a packet from its neighbor *l* (from *l*’s neighbor *p*), *i.e.*, *Pkt_l,p_*, then *i* enters the “Quiet” state. In the “Quiet” state, *i* keeps quiet to avoid interfering with neighbors. After a period of quiet time, *i* enters the “Idle” state. If *i* receives *RTS_k,i_*, then it transitions from the “Idle” state to the “Checking Scheduling” state. In the “Checking Scheduling” state, *i* determines whether the request conflicts with its schedule. If yes, it transfers to the “Idle” state and ignores the request. If the request does not conflict, *i* sends *CTS_i,k_* and enters the “Waiting Data” state. Sensor *i* transfers from the “Waiting Data” state to the “Checking Data” state after receiving *Data_k,i_*. Then, after the “Checking Data” state, the state is changed to “Idle” after *i* sends *Ack_i,k_*. Finally, if *i* intends to transmit to its neighbor *j*, *i* enters the “Waiting CTS” state after it sends *RTS_i,j_*. Sensor *i* enters the “Waiting Ack” after it receives *CTS_j,i_* from *j*. Sensor *i* then transmits *Data_i,j_*. While in the “Waiting Ack” state, *i* waits for *Ack_j,i_*. After receiving *Ack_j,i_*, *i* enters the “Idle” state. However, while waiting for *CTS_j,i_* during the “Waiting CTS” state, if sensor *i* receives another packet from *j*, e.g., *RTS_j,k_* or *CTS_j,k_*, then *i* enters the “Asking Extra Commu” state. In the “Asking Extra Commu” state, *i* requests an extra chance to communicate with *j* by exploiting the waiting resources. Moreover, while *i* receives *EXR_l,i_* during either the “Waiting CTS” or “Waiting Data” states, if *i* receives another packet from another neighbor, then it enters the “Asked Extra Commu” state. Note that *EXR_l,i_* is an extra RTS packet that *l* sends to *i*. We focus on the “Asking Extra Commu” and “Asked Extra Commu” states, which indicate a sensor is currently asking or has asked for an extra communication opportunity.

As described in Section 3.2, sensor *i* is the contention failed sensor, *j* is the sensor that *i* intends to communicate with, and *k* will communicate with *j*. Moreover, *l* represents another neighbor of *i*, and *p* is a neighbor of *l*. Furthermore, EXR and EXC are the extra negotiation packets. Note that EXR is an extra RTS packet and EXC is an extra CTS packet. In EW-MAC, RTS, CTS, Data, and Ack packets are sent at the beginning of a time slot; however, EXR, EXC, EXData, and EXAck packets are usually not.

### 4.2. Extra Communications for Negotiated Neighbors

Extra communication can be divided into request and transmission phases. The request phase includes sensor *i* sending EXR to and receiving EXC from *j*. The extra transmission phase includes *i* sending EXData to and receiving EXAck from *j*. Assume that sensor *i* sends *RTS_i,j_* to *j* but receives *CTS_j,k_* or *RTS_j,k_* from *j*. Then, sensor *i* asks for an extra chance to communicate if it is sure that the extra communications will not interfere with other predictable packets, *i.e.*, *Data_k,j_* or *CTS_k,j_*. If sensor *j* is a receiver in another negotiated communication, *i* sends the extra request after *j* sends *CTS_j,k_* and before it receives *Data_k,j_*. Thus, the extra request exploits time periods V of sensor *j* and VII of sensor *i* (Figure 2). In contrast, if *j* is a sender in another negotiated communication, *i* sends the extra request after *j* sends *RTS_j,k_* and before it receives *CTS_k,j_*. Thus, the extra request exploits time periods III of sensor *j* and I of sensor *i* (Figure 2).

Furthermore, when sensor *i* requests extra communication, it must consider its other neighbors. If any neighbor of *i* sends RTS before *i* in the previous time slot, this prevents *i* from sending *RTS_i,j_*. Hence, the other negotiated neighbors of *i* must be receivers. Therefore, *i* should ensure that *EXR_i,j_* arrives at those neighbors in period V. If sensor *i* receives *EXC_j,i_* after twice the propagation time, then *i* and *j* proceed to the extra transmitting phase. Otherwise, *i* gives up the extra transmission and returns to the “Quiet” state (Figure 3).

When sensors *i* and *j* are in the extra transmitting phase, *i* transmits *EXData_i,j_* when other neighbor sensors are not currently receiving data or sending Ack packets. If sensor *j* is a receiver in other communication, *i* sends *EXData_i,j_* in the period after *j* sends *Ack_j,k_* and receives *Data_k,j_*. In addition, *EXData_i,j_* and *EXAck_j,i_* exploit time periods VI of sensor *j* and VII of sensor *i* (Figure 3). In contrast, if *j* is a sender in other communication, *i* sends *EXData_i,j_* in the period after *j* sends *Ack_j,k_*. In other words, the time for *EXData_i,j_* arriving at sensor *j* is after it sends *Ack_j,k_*. Here, *EXData_i,j_* and *EXAck_j,i_* exploit time periods IV of sensor *j* and I and II of sensor *i* (Figure 2). Moreover, when other neighbors are communicating, the extra communication cannot interfere with their communication. Thus, sensor *i* should ensure that *EXData_i,j_* arrives at the other neighbors in the period IV after they send Ack packets.

After receiving *EXData_i,j_*, an *EXAck_j,i_* packet is transmitted by sensor *j*. Then, the extra communication between *i* and *j* is completed. Note that when a sensor receives any extra control packet from its neighbor, its original negotiated communication must not be interfered with and the sensor will be quiet to avoid interfering with the extra communication after it completes its negotiated communication.

**Figure 4:**
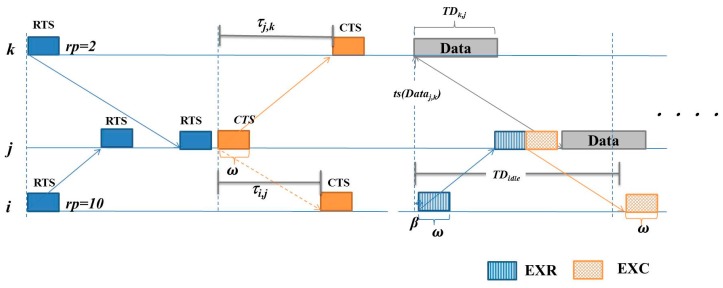
Sensor *i* asking an extra communicating chance to sensor *j*.

Following the description abovementioned, an example is presented for showing the detail. Figure 4 is the example in a simple case. Sensors *i* and *k* send RTSs, each including sender ID, sending time stamp, and the random priority value, *rp*, to *j*. Sensor *j* may choose sensor *k* which with higher *rp* to communicate with. Thus, *j* transmits *CTS_j,k_* which includes the *τ_j,k_* and its sending time stamp to announce its choice. After receiving the *CTS_j,k_*, sensor *i* knows the idle time of *j* and the propagation delay between sensors *i* and *j* as well as between sensors *j* and *k*. Moreover, if the idle duration and propagation delay among *i*, *j* and *k* satisfied Equation (6), then *i* transmits *EXR_i,j_* in the next time slot of *CTS_j,k_* at the beginning after *β*. When *j* receives the *EXR_i,j_*, it transmits an *EXC_j,i_* to notify that sensor *i* is allowed to transmit the EXData if the extra transmission will not interfere *j*’s negotiated communication.

**Figure 5:**
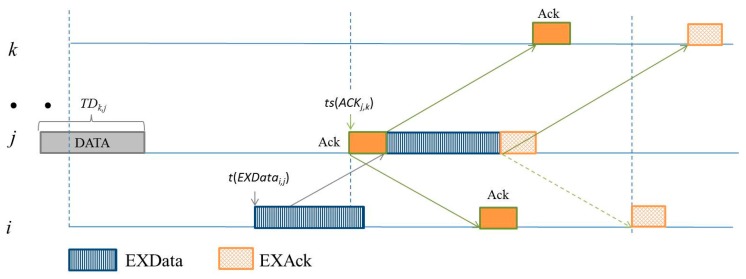
Sensor *i* transmits EXData to sensor *j.*

After receiving *EXC_j,i_*, to prevent the negotiated communications, sensor *i* transmits *EXDATA_i,j_* avoiding the time for *j* receiving *Data_k,j_* and sending *Ack_j,k_*. Thus, *i* will transmit *EXData_i,j_* and the *EXData_i,j_* should arrive at *j* after *j* sending out *Ack_j,k_*. Let *t*(*Pkt*) represent the starting time for transmitting *Pkt* packet. The transmission time can be derived by Equation (6),

*t*(*EXData_i,j_*) = *ts*(*Ack_j,k_*) × (*ω* + *τ_max_*) + *ω* − *τ_i,j_* (6)

which leads *EXData_i,j_* arriving at *j* after *Data_k,j_* receiving and *Ack_j,k_* transmitting*.* In Equation (6), *ω* and *ω + τ_max_* represent the time length for transmitting a control packet and the length of a time slot, respectively. The time slot of *Ack_j,k_* can be obtained by Equation (5). Figure 5 shows the example following Figure 4. Sensor *i* transmits *EXData_i,j_* which arrives at sensor *j* after *j* sends *Ack_j,k_* to sensor *k*. In this case, the proposed protocol exploits the time periods V, VI, and VII of sensors as shown in Figure 2.

### 4.3. Initialization and Information Maintenance 

To determine accurate sending time for sensors, the propagation delay between neighbors should be known and maintained. To decrease the amount of maintained and transmitted information, sensors in the proposed algorithm maintain the propagation delay information for all one-hop neighbors. When sensor sensors are deployed, each sends a Hello packet that includes its ID and timestamp. As mentioned previously, the proposed protocol assumes that all the sensors in the network are synchronized. Thus, each sensor knows the propagation delay of all its neighbors after it receives the timestamps from neighbor packets. To avoid interfering with communicating neighbors, the propagation information of one-hop neighbors should be maintained by each sensor. Since the distance between two sensors may change, broadcasting one-hop neighbors to help sensors maintain two-hop neighbors is inefficient. Neither saving the information of two-hop neighbors nor broadcasting the information of a sensor’s one-hop neighbors is required in EW-MAC. In EW-MAC, each sensor maintains the propagation delay of its one-hop neighbors by adjusting whenever it receives packets from its neighbors. Therefore, to maintain the information and avoid interference by other sensors, the timestamp and propagation delay with communicating neighbors are added to all packets. Since a sensor receives the propagation delay of its neighbors, each sensor can send an extra request without interfering with its one- and two-hop neighbors.

To summarize the proposed EW-MAC protocol, if a sensor receives neighbor negotiation packets before it sends an RTS packet, it will not send the RTS packet. Only after it has sent the RTS to its neighbor, it may request extra communication when it fails to contend for transmission. Note that the extra communication must not interfere with negotiated communications. Each sensor maintains the propagation delay of its one-hop neighbors. The information of two-hop neighbors is not maintained, and communicating neighbors will announce the information if they are negotiated. With EW-MAC, resources have higher utilization because it exploits idle resources. Furthermore, data packets are not bound by a fixed data size.

## 5. Analysis and Performance Evaluation

Here, we discuss simulation and analysis of the performance of the proposed protocol against the S-FAMA [3], ROPA [4], and CS-MAC [18] protocols in terms of throughput and bandwidth utilization. S-FAMA is a slotted protocol that divides time into small time slots. To avoid interference, the duration of each time slot is equal to the maximal propagation delay period. Each data communication is negotiated by sending control packets including RTS and CTS at the beginning of a time slot. For example, when sensor *a* intends to transmit data to *b*, *a* will send an RTS to *b* at the beginning of the *t*th time slot. When sensor *b* is able to receive data from *a*, *b* returns a CTS at the beginning of the (*t* + 1)th slot; thus, sensor *a* can transmit data to *b* at the beginning of the (*t* + 2)th time slot. When the neighbors receive the control packet in the *t*th or (*t* + 1)th slot, they will not interfere with communication between sensors *a* and *b*. With ROPA, each sender sends the RTS packet including the propagation delay time between the sender and receiver. If a neighbor of the sender intends to communicate with the sender, then the neighbor can send an RTA packet, *i.e.*, extra RTS, during the wait time of the sender if the RTA packet does not interfere with the arrival of the CTS packet. With CS-MAC, a neighbor forces utilization of the waiting resources by directly sending data packets when it knows the wait time is sufficient. The topology of the simulation environment is deployed as Figure 1. Each sensor receives packets from sensors in greater depths. In addition, each sensor transmits packets to sensors closer to the surface.

This section examines the performance of the proposed protocol against the existing protocols in 1000 km^3^ underwater environments with 60 or more distributed sensor sensors. Because the simulator should consider underwater propagation, we employ the UAN propagation model of NS-3. For accurately verifying the mechanisms, we select the Bellhop Propagation for channel model. Moreover, the Default PER model and Default SINR are chosen for PHY model. Furthermore, we rewrite the MAC model based on CW-MAC which is a slotted contention MAC protocol. The simulator produces implementations by referencing the equations in Section 2. In our simulations, the location models include non-moved, moved horizontal, or moved vertical. The location of each sensor is changed by randomly selecting one of these models. The propagation delays used in Bellhop propagation model are varied according the changed locations. To transmit data to the surface sink, sensors at greater depths must transmit packets to sensors closer to the surface. The transmission range and speed are 1.5 km and 1.5 km/s, respectively. The bandwidth of the network is 12 kbps. The size of the control packets including RTS, CTS, and Ack is 64 bits. This work considers simulations with different data packet sizes (1024–4096 bits). Unless otherwise stated, the data packet size is set to 2048 bits. The simulation parameters are listed in Table 2.

### 5.1. Throughput

**Figure 6:**
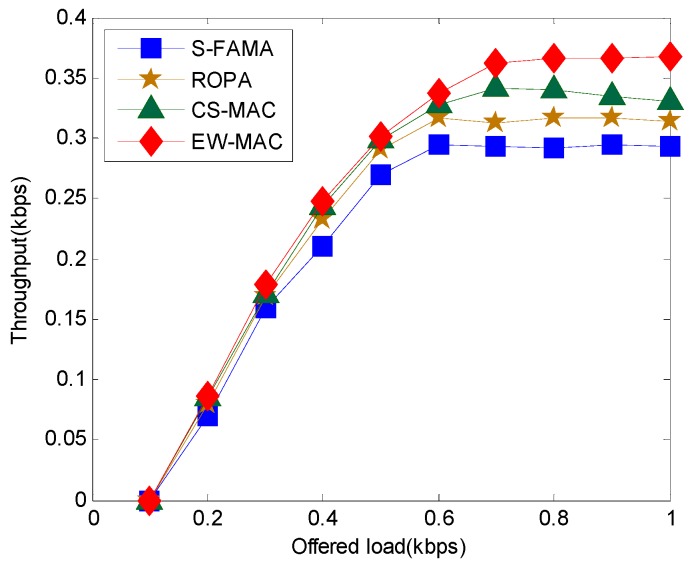
Throughput at different offer loads.

First, we evaluate the throughput of the proposed protocol against S-FAMA, ROPA, and CS-MAC. Figure 6 shows that the throughput of EW-MAC is greater than that of CS-MAC, ROPA, and S-FAMA. Throughput increases when the offer load (or traffic load) increases from 0.1 to 0.6 kbps. Since ROPA exploits the sender’s idle bandwidth, the throughput of ROPA is better than that of S-FAMA. Moreover, since CS-MAC and EW-MAC exploit the idle bandwidth of both senders and receivers, the throughput of CS-MAC and EW-MAC are better than that of ROPA. CS-MAC is better than EW-MAC when the offered load is less than 0.6 because it directly transmits data without extra negotiation. However, when the offer load is greater than 0.6, CS-MAC throughput is much less than EW-MAC. A higher offer load indicates that more data must be transmitted. CS-MAC exploits the wait time of sensors without assessing how transmission will interfere with other neighbors; thus, additional transmission will increase the interference effect. Therefore, the throughput of CS-MAC decreases when the offer load is greater than 0.8.

**Figure 7:**
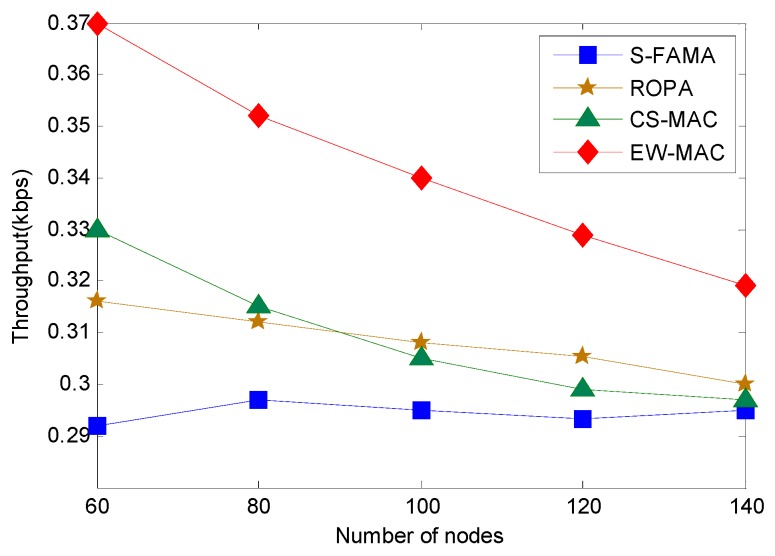
Throughput at different network sensor densities.

Here, we evaluate the relationship between network environment and throughput when the offer load is 0.8 kbps. As shown in Figure 7, increasing sensor density will reduce propagation delay between sensors. When sensor density is high, the wait time that can be exploited is less. Thus, protocols that exploit idle time demonstrate decreased performance. In particular, since CS-MAC determines transmission by the wait time of sensors and the transmitting time for data packets, the reduced waiting resources will reduce the chance to perform extra transmissions. Thus, throughput is less when sensor density is high. For S-FAMA, each transmission reserves a maximal propagation delay; thus, varying sensor density does not affect performance. Since EW-MAC exploits the wait time between sending a packet and the next packet arriving, propagation delay is reduced when sensor density is high. Thus, the chance of transmitting the extra packet is reduced. Therefore, the worst cases of EW-MAC, CS-MAC, and ROPA have the same throughput as S-FAMA, *i.e.*, no extra communication pair is added to the network.

**Figure 8:**
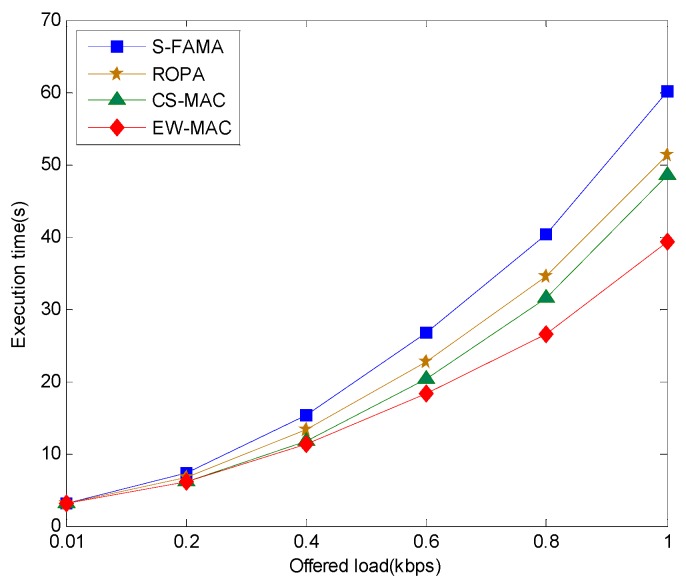
Relationship between execution time and offer load.

The time for successful transmission is another important index. Figure 8 shows that when the number of transmitting packets is less than 20 per 300 s, *i.e.*, offer load of approximately 0.136, the execution time differences are not significant. However, the greater the offer load, the greater the difference. Using waiting resources, CS-MAC, ROPA, and EW-MAC can perform those transmissions faster. ROPA uses only sender resources; thus, execution time is greater than those of CS-MAC and EW-MAC with the same offered load. Since the extra transmission packet is bound without extra negotiation, successful extra transmissions in CS-MAC are fewer than those in EW-MAC. Thus, EW-MAC is better than CS-MAC, CS-MAC is better than ROPA, and ROPA is better than S-FAMA.

### 5.2. Power Consumption

**Figure 9:**
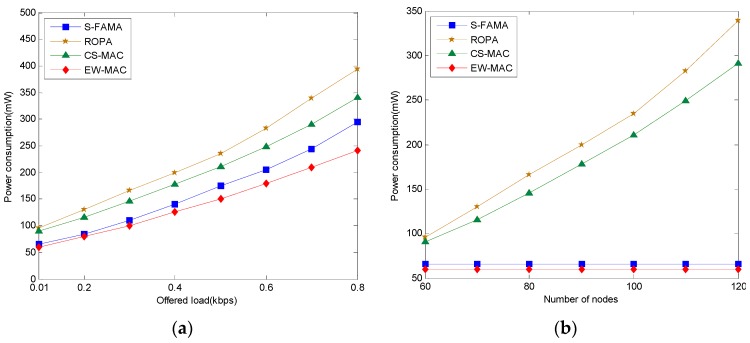
Power consumption. (**a**) The protocols consume energy according to the offered load within range (0.1, 0.8) k bytes in each second among 80 sensors; (**b**) The protocols consume energy according to the number of sensors within range (60, 100) while offered load is 0.3 kbps.

Here, we evaluate power consumption including the power for waiting, transmitting, and receiving a number of transmissions. We compare the power consumption of algorithms when they transmit varied amounts of information. Since ROPA and CS-MAC must maintain and transmit two-hop neighbor information, they consume more energy. Since EW-MAC does not maintain the two-hop neighbor information and transmits faster, its power consumption is much less than that of S-FAMA. Considering the energy consumption required for waiting, transmitting, and maintaining, ROPA consumes more energy than CS-MAC and CS-MAC consumes more energy than S-FAMA. By knowing the distance between one-hop neighbors and exploiting idle time, the EW-MAC protocol communicates in less time with fewer transmissions. Thus, EW-MAC shows the best power consumption characteristics. On the other hand, when the same amount of information is transmitted, the numbers of transmissions with ROPA and CS-MAC are much greater when the number of sensors increases. However, S-FAMA and EW-MAC do not increase the number of transmissions according to the number of sensors. Therefore, S-FAMA and EW-MAC consume much less power than ROPA and CS-MAC when the number of sensors increases. The simulation results are shown in Figure 9.

### 5.3. Overhead

**Figure 10:**
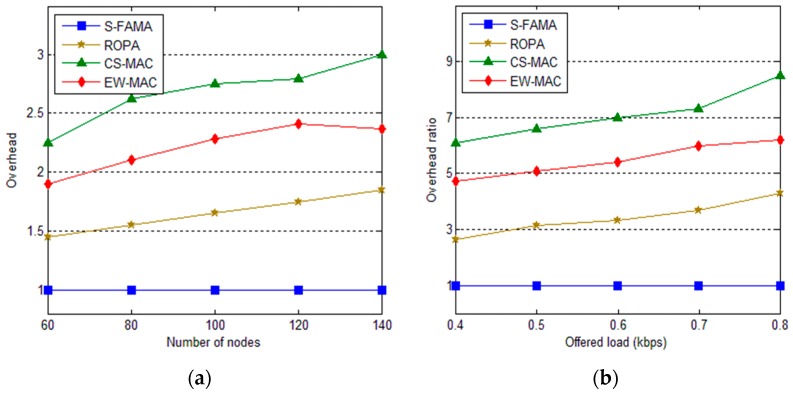
Overhead in different network situations. (**a**) The overhead of protocols spend for the number of sensors within range (60, 140) while offered load is 0.5kbps; (**b**)The overhead ratio of protocols according to the offered load within range (0.4, 0.8) kbps among 200 sensors.

Here, we investigate overhead. To utilize idle bandwidth without interfering with other communications, additional computations, transmissions, and maintenance are required. An increased amount of sensors implies more communications and more complicated computations. Moreover, since each sensor should maintain neighbor information, additional memory is required. Thus, memory requirements depend on the amount and complexity of the computations and the number of neighbors. Since S-FAMA does not require additional computation or storage, S-FAMA resources are considered as the baseline. The overhead values are calculated by comparing transmission cost, cost of maintaining neighbors, and retransmission cost of S-FAMA. Retransmissions occur when transmissions collide. The neighbor maintenance cost includes the cost of accessing neighboring information, carrying more information as piggyback, and transmitting messages without piggyback. Since EW-MAC, CS-MAC, and ROPA require the propagation delay of one-hop neighbors, additional initialization costs are required. Furthermore, control packets include the extra one-hop neighbor information. Since ROPA has less chance for communication, extra resource consumption is less than that of CS-MAC and EW-MAC. In addition, since CS-MAC control packets include two-hop neighbor information, its overhead is much greater than that of EW-MAC, which includes only one-hop neighbor information. As shown in Figure 10, ROPA overhead is 1.5 times that of S-FAMA on average. In addition, CS-MAC and EW-MAC demonstrate more overhead than S-FAMA and ROPA (generally, 2–3 times the overhead of S-FAMA). As shown in Figure 10a, when the offered load is large, more communication and overhead are required. However, by evaluating overhead according to the number of sensors, ROPA and CS-MAC must periodically transmit two-hop neighbor information. With EW-MAC, only one-hop neighbor information is transmitted; thus, its overhead does not increase as much as it does for ROPA and CS-MAC.

**Figure 11:**
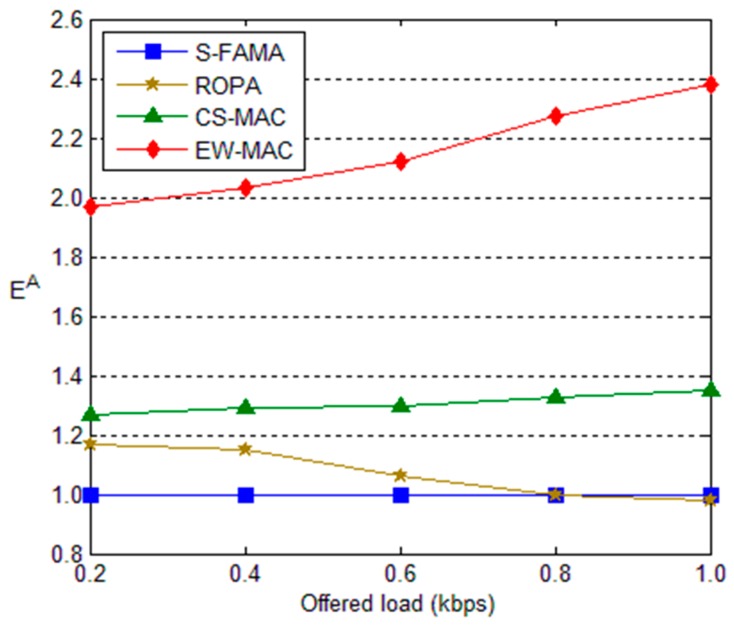
Efficiency indexes for different offered loads.

Finally, we evaluate the efficiency index of each protocol. Here, we set the index of S-FAMA as the basis, *i.e.*, 1. The efficiency indexes of the other protocols are shown by the y-axis in Figure 11. As defined in Equation (4), the efficiency index increases in proportion to throughput and bandwidth utilization but decreases in proportion to the ratio of power consumption. Although the power consumption of ROPA, CS-MAC, and EW-MAC is greater than that of S-FAMA, the efficiency indexes of these protocols are typically greater than that of S-FAMA due to higher throughput and bandwidth utilization. However, when the offered load is greater than 0.8, more serious interference reduces the throughput of ROPA. Thus, the efficiency index of ROPA is less than that of S-FAMA. As seen in Figure 10 and Figure 11, both overhead and efficiency increase when the offered load increases. Moreover, the overhead increase is much more than increase of the efficiency index when the offered load increases, especially when the offered load is greater than 0.7.

Because the underwater scenario is variable, the sound speed and transmission distance among sensors are changeable according to the locations of changed sensors. Hence, the *τ*_max_ may be changed. To be sure of collision free by slotted design, the time length of a slot is set to the maximum length for transmission in the environment. However, the varied propagation delays among sensors impact our protocol seriously. If the related distance between each pair of sensors is changeable, then the maintained propagation delay information is useless for predicting the arriving time of packets. Therefore, the protocol is appropriate while applying in an environment with stable relation among sensors. If the relations among sensors are changeable shortly, the proposed protocol is not applying well.

## 6. Conclusions

This study proposed a slotted protocol to improve throughput and reduce energy consumption in UASNs. Since the propagation delay in UASNs is significant, the proposed protocol exploits the idle bandwidth to increase the chance of extra communications that have failed in previous contention. The extra communications are allowed if they will not interfere with currently communicating pairs of sensors. The extra negotiations can utilize the resources of receivers during the period after sending CTSs and before receiving data or utilize the resources of senders during the period after sending RTSs and before receiving CTSs. After extra communications are successfully negotiated, the extra transmission is allowed. By parallel transmissions with limited bandwidth, bandwidth utilization and throughput of the network are improved. By decreasing the wait time, the energy consumption required for the same amount of communication is reduced. Our performance evaluation demonstrates that the proposed protocol outperforms S-FAMA, ROPA, and CS-MAC in terms of throughput, bandwidth utilization, and energy efficiency, especially when the data packet size is large or sensor deployment is dense.

## Figures and Tables

**Table 1:** Notations.

Notation	Description
$dr_{k}^{T}$	Number of packets *k* receives successfully during period *T*
*Pkt_s,r_*	A packet whose sender is *s* and destination is *r*, *Pkt* can be RTS, CTS, Data, Ack, EXR (extra RTS), EXC (extra CTS), EXData (extra Data), or EXAck (extra Ack)
*Pkt^t^_s,r_*	Boolean value; if *Pkt_s,r_* is transmitted at time *t*, then *Pkt^t^_s,r_* = 1, otherwise *Pkt^t^_s,r_* = 0
*|Pkt^t^_s,r_*|	Duration *s* transmits *Pkt^t^_s,r_*
*ts*(*Pkt^t^_s,r_*)	Time slot in which *Pkt^t^_s,r_* is transmitted
*N*(*x*)	Set of sensors that are one-hop neighbors of sensor *x*
*τ_xy_*	Propagation delay between sensors *x* and *y*
*ω*	Duration of a transmitted or received control packet
*|ts|*	Duration of a time slot

**Table 2:** Simulation parameters.

Parameter	Value
Number of sensors	60
Deployment area	1000 km^3^
Bandwidth	12 kbps
Communication range	1.5 km
Acoustic transmission speed	1.5 km/s
Simulation time	300 s
Control packet size	64 bits
Data packet size	1024–4096 bits
